# The Complex Interplay Between Depression/Anxiety and Executive Functioning: Insights From the ECAS in a Large ALS Population

**DOI:** 10.3389/fpsyg.2018.00450

**Published:** 2018-04-05

**Authors:** Laura Carelli, Federica Solca, Andrea Faini, Fabiana Madotto, Annalisa Lafronza, Alessia Monti, Stefano Zago, Alberto Doretti, Andrea Ciammola, Nicola Ticozzi, Vincenzo Silani, Barbara Poletti

**Affiliations:** ^1^Laboratory of Neuroscience, Department of Neurology, Istituto Auxologico Italiano – Istituto di Ricovero e Cura a Carattere Scientifico, Milan, Italy; ^2^Department of Pathophysiology and Transplantation, “Dino Ferrari” Center, Università degli Studi di Milano, Milan, Italy; ^3^Department of Cardiovascular, Neural and Metabolic Sciences, Istituto Auxologico Italiano – Istituto di Ricovero e Cura a Carattere Scientifico, Milan, Italy; ^4^Research Centre on Public Health, Department of Medicine and Surgery, University of Milano-Bicocca, Milan, Italy; ^5^Department of Neurorehabilitation Sciences, Casa di Cura Privata del Policlinico, Milan, Italy; ^6^Department of Neuroscience and Mental Health, IRCCS Fondazione Ca’ Granda Ospedale Maggiore Policlinico, Università degli Studi di Milano, Milan, Italy

**Keywords:** depression, anxiety, executive functions, social cognition, ECAS, amyotrophic lateral sclerosis

## Abstract

**Introduction:** The observed association between depressive symptoms and cognitive performances has not been previously clarified in patients with amyotrophic lateral sclerosis (pALS). In fact, the use of cognitive measures often not accommodating for motor disability has led to heterogeneous and not conclusive findings about this issue. The aim of the present study was to evaluate the relationship between cognitive and depressive/anxiety symptoms by means of the recently developed Edinburgh Cognitive and Behavioral ALS Screen (ECAS), a brief assessment specifically designed for pALS.

**Methods:** Sample included 168 pALS (114 males, 54 females); they were administered two standard cognitive screening tools (FAB; MoCA) and the ECAS, assessing different cognitive domains, including ALS-specific (executive functions, verbal fluency, and language tests) and ALS non-specific subtests (memory and visuospatial tests). Two psychological questionnaires for depression and anxiety (BDI; STAI/Y) were also administered to patients. Pearson’s correlation coefficient was used to assess the degree of association between cognitive and psychological measures.

**Results:** Depression assessment negatively correlated with the ECAS, more significantly with regard to the executive functions subdomain. In particular, Sentence Completion and Social Cognition subscores were negatively associated with depression levels measured by BDI total score and Somatic-Performance symptoms subscore. Conversely, no significant correlations were observed between depression level and cognitive functions as measured by traditional screening tools for frontal abilities (FAB) and global cognition (MoCA) assessment. Finally, no significant correlations were observed between state/trait anxiety and the ECAS.

**Discussion and conclusion:** This represents the first study focusing on the relationship between cognitive and psychological components in pALS by means of the ECAS, the current gold standard for ALS cognitive-behavioral assessment. If confirmed by further investigations, the observed association between depression and executive functions suggests the need for a careful screening and treatment of depression, to avoid overestimation of cognitive involvement and possibly improve cognitive performances in ALS.

## Introduction

A consistent body of literature concerning cognitive-behavioral alterations in amyotrophic lateral sclerosis (ALS) in the last 20 years allowed to recognize it as a multisystem disorder, and not a purely motor neuron disease. In particular, specific cognitive alterations have been described, together with psychological and behavioral changes, with underlying uncompletely determined patterns of neuroradiological, neurobiological, and genetic profiles (DeJesus-Hernandez et al., 2011; Phukan et al., 2012; Goldstein and Abrahams, 2013; Agosta et al., 2016). A prevalence of 30–50% of cognitive impairment, predominantly in the form of executive dysfunction, has been described (Phukan et al., 2012; Montuschi et al., 2015); in about 10–15% of ALS patients these changes fulfill the criteria for frontotemporal dementia – FTD (Phukan et al., 2012; Goldstein and Abrahams, 2013; Montuschi et al., 2015). Prevalence rates for depression and anxiety in ALS range from 0 to 44% and from 0 to 30%, respectively (Kurt et al., 2007). Both psychological aspects and cognitive changes exert a well-known effect on quality of life, functional abilities, prognosis and survival of patients with ALS (pALS) (Elamin et al., 2013; van Groenestijn et al., 2016; Xu et al., 2017). An association between cognitive deficits and depression has been consistently observed in both clinical and medically healthy populations (Lim et al., 2013; Rock et al., 2014; Pu et al., 2017; Yoon et al., 2017). In particular, depressive symptoms seem to primarily affect executive functioning, with neuroimaging findings supporting the involvement of brain networks entailing frontal-subcortical circuits in depressed patients (Rock et al., 2014; Ahern and Semkovska, 2016; Brakowski et al., 2017).

Despite the presence of consistent evidence in other neurological diseases (e.g., Terroni et al., 2012; Nunnari et al., 2015; Snowden et al., 2015), the association between cognition and psychological aspects has been poorly investigated in ALS, also showing conflicting results. A recent study revealed the absence of a relation between cognitive impairment and psychiatric/psychosocial measures, as well as between cognitive impairment and wish to die, employing a neuropsychological assessment focused on executive functions (Rabkin et al., 2016). On the contrary, other studies adopting different measures of cognitive functions showed an association between psychological/psychiatric symptoms and global cognition (Wei et al., 2016) and between depression and specific neuropsychological aspects, including verbal and visual learning, processing speed and language (Jelsone-Swain et al., 2012).

When considering more innovative cognitive tools, two recent studies about feasibility of Eye-Tracking (ET) and Brain Computer Interface (BCI) technologies for cognitive testing in ALS highlighted a negative correlation between anxiety levels and reasoning time at some neuropsychological tests for executive functions. Such results were interpreted according to an increase in the rate of impulsivity, possibly depending both on the reduced amount of cognitive and inhibitory resources in pALS and on the supplementary influence of anxiety on such cognitive profile (Poletti et al., 2016a,b). In both studies, no significant correlations were observed between ET tests and depression level, as measured by Beck Depression Inventory (Beck et al., 1961).

A relevant issue in the neuropsychological assessment of pALS concerns the absence of validated gold standard tools, accommodating for progressive physical disabilities and providing reliable, comprehensive and comparable results across studies. This aspect remained an unsolved issue till the recent development (Abrahams et al., 2014), and validation into several languages (Lulé et al., 2015; Niven et al., 2015; Poletti et al., 2016c) of a rapid multi-domain cognitive-behavioral screening tool specifically designed for ALS, i.e., the Edinburgh Cognitive and Behavioral ALS Screen – ECAS. Moreover, the ECAS has been specifically designed to accommodate for verbal/motor disability, since subtests can be administered both in spoken and written form and in moderate/advanced stages of the disease. Despite the rapid and large diffusion of such screening tool for the clinical assessment of pALS, it has never been applied in order to specifically investigate the association between cognitive and psychological aspects. Only two studies report the use of the ECAS in association to psychological and behavioral scales, without specifically addressing such topic. In particular, Radakovic et al. (2017) described the absence of correlation between depression evaluated by means of Geriatric Depression Scale and ‘cognitive functioning task performances’ including the Verbal Fluency Total Score of the ECAS. Another study (Niven et al., 2015) described the absence of correlations between anxiety and ECAS scores, but no indications were provided about symptoms of depression.

In the context of the validation of the Italian ECAS, we preliminarily explored the relationship between such instrument and psychological variables such as anxiety and depression (Poletti et al., 2016c). In the recruited sample, a mild negative association was observed between depression assessment and both global and ALS-specific functions scores at the ECAS; moreover, state anxiety assessment negatively correlated with the ECAS total and the ALS non-specific functions scores.

As above described, previous literature data on other medically ill and non-medically ill populations (Terroni et al., 2012; Lim et al., 2013; Rock et al., 2014; Nunnari et al., 2015; Snowden et al., 2015; Pu et al., 2017; Yoon et al., 2017) supports the presence of a relationship between a psychological aspect, i.e., presence/severity of depressive symptoms and cognitive profiles. Basing on such evidence, as well as on conflicting results regarding pALS and on our preliminary findings, we aimed to investigate the association between psychological features and cognitive abilities with the ECAS in a larger ALS population, particularly focusing on executive functions. Such association was also investigated with traditional cognitive measures of frontal (FAB) and global cognitive functioning (MoCA) not specifically designed for ALS, in order to identify possible differences between the gold standard ALS instrument and traditional cognitive tools for such purposes.

The presented research is part of a larger and ongoing clinical study using the ECAS for longitudinal assessment, evaluating its feasibility and sensitivity across the course of ALS disease.

## Materials and Methods

### Participants

Sample included 168 ALS patients (Males: 114; Females: 54; age: 62.3 ± 12.1 years; education: 11.1 ± 4.4 years; disease duration: 33.6 ± 42.7 months) recruited at the Department of Neurology, IRCCS Istituto Auxologico Italiano. The diagnosis of ALS was made by neurologists experienced in the field of neuromuscular diseases, with patients fulfilling the revised El Escorial criteria for clinically possible, probable, probable – laboratory-supported or definite ALS (Brooks et al., 2000). Patients in terminal stages of disease or with major comorbid medical, neurological or cardio-vascular diseases were excluded from the study. Disease status was evaluated using the ALS Functional Rating Scale-Revised – ALSFRS-R (Cedarbaum et al., 1999). The study protocol was reviewed and approved by the Ethics Committee of our Institution and all eligible subjects received verbal and written information about the study. All participants signed an informed consent, according to the Declaration of Helsinki. Patients performed the designed cognitive and psychological protocol, as described below; it was administered by trained neuropsychologists and required approximately 40 min.

### Cognitive and Psychological Assessment

#### Cognitive Assessment

A neuropsychological and psychological protocol employed in a previous study (Poletti et al., 2016c) was adopted, including both two standard cognitive measures and a recently validated rapid cognitive-behavioral screening tool specifically developed for ALS (Edinburgh Cognitive and Behavioral ALS Screen – ECAS).

The Italian version of the ECAS was administered, assessing different cognitive domains, including ALS-specific (executive functions, fluency and language tests) and ALS non-specific domains (memory and visuospatial functions tests). Each domain includes specific tests (see **Table 1**). Moreover, it involves a separate Carer Interview, investigating behavior and psychotic alterations.

**Table 1:** pALS’ scores at the standard cognitive screening and the ECAS.

	Mean ±*SD*	Range	Cut-off
FAB (*n* = 149)	15.81 ± 1.68	10–18	13.4
MoCA (*n* = 143)	22.78 ± 2.86	14–30	17.36
ECAS total score (*n* = 168)	99.67 ± 18.73	31–129	97
ALS specific functions	73.24 ± 15.12	21–97	71
Language functions	23.30 ± 4.19	9–28	22
Naming	6.64 ± 1.59	0–8	–
Comprehension	7.57 ± 0.83	4–8	–
Spelling	9.09 ± 2.83	0–12	–
Fluency	16.01 ± 5.75	0–24	14
Fluency letter S	8.39 ± 3.01	0–12	–
Fluency letter C	7.62 ± 3.57	0–12	–
Executive functions	33.93 ± 7.59	9–47	31
Reverse digit span	5.32 ± 1.87	0–11	–
Alternation	9.70 ± 3.36	0–12	–
Sentence completion	9.36 ± 2.56	0–12	–
Social cognition	9.55 ± 2.97	1–12	–
ALS non-specific functions	26.43 ± 5.00	10–34	24
Memory functions	15.05 ± 4.59	1–22	13
Immediate recall	5.24 ± 2.10	0–9	–
Delayed retention	7.69 ± 2.68	0–10	–
Delayed recognition	2.11 ± 1.26	0–4	–
Visuospatial functions	11.38 ± 1.02	6–12	10
Dot counting	3.90 ± 0.30	3–4	–
Cube counting	3.58 ± 0.80	1–4	–
Number location	3.90 ± 0.39	1–4	–


*Data are expressed as Means ± SD or absolute numbers*.

Standard cognitive measures included two brief batteries; for frontal executive functions, Frontal Assessment Battery – FAB (Dubois et al., 2000) was employed, evaluating subdomains of conceptualization, mental flexibility, motor programming, sensitivity to interference, inhibitory control, and environmental autonomy; for global cognitive functioning, Montreal Cognitive Assessment – MoCA (Italian translation by Pirani et al., 2007; Italian normative data by Conti et al., 2014) was administered.

#### Psychological Assessment

The evaluation of depressive and anxiety symptoms was performed by means of two self-rated measures widely used in ALS, i.e., the *Beck Depression Inventory* (BDI) (Beck et al., 1961) and *State-Trait Anxiety Inventory-Y* (STAI-Y) (Spielberger et al., 1970), for both state (STAI-Y1) and trait (STAI-Y2) anxiety components assessment. The BDI consists of 21 items, concerning both cognitive-affective (BDI CA, items 0–13) and somatic-performance (BDI SP, items 14–21) symptoms of depression. Total score ranges from 0 to 63, with higher total scores indicating more severe depressive symptoms. The standard cut-off ranges are as follows: 0–9 indicates minimal depression, 10–18 indicates mild depression, 19–29 indicates moderate depression, 30–63 indicates severe depression. The 40-item STAI-Y scores range from 20 to 80. Questions refer to how anxious people are feeling at the time of the study (state) and in general (trait). Scores higher than 65 indicate a clinically relevant anxiety.

### Statistical Analysis

Descriptive statistics (mean ± standard deviations for continuous variables and absolute number and frequencies for discrete variables) were used to describe the main characteristics of our sample and performances obtained at the ECAS and at standard cognitive/psychological assessments. *Pearson’s correlation coefficient* was used to assess the degree of association between measures. An α level of 0.05 was used for all hypothesis tests. *P*-values were adjusted with a ‘False Discovery rate’ approach for multiple comparison correction (Benjamini and Hochberg, 1995). All data analyses were performed using SAS 9.2 software (SAS Institute, Cary, NC, United States).

## Results

### Patients’ Demographic and Clinical Characteristics

Clinical neurological examination showed a mean ALSFRS/R score of 37.2 ± 7.5 (ALSFRS/R Bulbar: 10.2 ± 2.3); 51 patients presented upper limb regions involvement at onset, 70 lower limb regions, 9 upper and lower limb, 35 bulbar, 2 bulbar and lower limb and one respiratory symptoms at onset. Ten patients had non-invasive ventilation (NIV) and no one had percutaneous endoscopic gastrostomy (PEG). For description of patients’ scores at ECAS and standard cognitive screening measures, see **Table 1**.

With regard to psychological aspects, of the 154 patients who completed the BDI, one hundred (65%) showed a clinically significant depression, ranging from mild-to-moderate (43%), moderate-to-severe (17%) and severe (5%). Of the 155 patients who completed the STAI-Y, 13 (8%) showed a clinically significant state anxiety and 15 (10%) showed relevant trait anxiety levels. See **Table 2** for description of patients’ scores at the psychological assessment.

**Table 2:** pALS’ scores at the psychological assessment.

	Mean ±*SD*	Range
**Beck Depression Inventory (BDI) (*n* = 154)**		
Total score	14.13 ± 8.84	1–58
Cognitive-affective symptoms	6.49 ± 5.95	0–41
Somatic-performance symptoms	7.64 ± 3.92	0–18
**State-Trait Anxiety Inventory (Form Y) (*n* = 155)**		
State anxiety – Y1 (*T*-score)	49.46 ± 9.47	34–76
Trait anxiety – Y2 (*T*-score)	50.92 ± 10.25	30–80


*Data are expressed as Means ± SD or absolute numbers.*

### Relationship of Psychological Symptoms to ECAS Scores and Subdomains

Analysis of association between cognitive and depression scores revealed the presence of significant, even if modest, correlations between variables. In particular, depression assessment negatively correlated with the ECAS Total and the ALS-Specific functions scores, mainly with regard to Somatic-Performance symptoms and BDI Total score (BDI SP-ECAS Total score: *r* = –0.21, *p* = 0.023; ALS-Specific functions score: *r* = –0.22, *p* = 0.019; BDI Total score-ECAS Total score: *r* = –0.22, *p* = 0.019; ALS-Specific functions score: *r* = –0.23, *p* = 0.019; BDI CA-ECAS Total score: *r* = –0.19, *p* = 0.028; ALS-Specific functions score: *r* = –0.19, *p* = 0.027). No significant correlations were observed between anxiety and the ECAS, concerning both Total score and ALS-non Specific/Specific functions.

Analysis performed on the ECAS subdomains revealed mild significant, negative correlations between the ECAS executive functions subdomain and both Cognitive-Affective and Somatic-Performance symptoms of depression (BDI CA-ECAS executive functions: *r* = –0.21, *p* = 0.023; BDI SP-ECAS executive functions: *r* = –0.24, *p* = 0.017). Moreover, negative correlations were also observed between BDI total score and Executive functions subdomains as well as Language (BDI Total score-ECAS Language: *r* = –0.20, *p* = 0.023; ECAS executive functions: *r* = –0.25, *p* = 0.016). Language subdomain also correlated with Cognitive-Affective BDI subscore (*r* = –0.19, *p* = 0.027).

#### Relationship of Psychological Symptoms to Specific Cognitive Tests of the ECAS Subdomains

Further analysis performed on cognitive tests included in the subdomains revealed that, with regard to executive functions subdomain, Sentence Completion and, particularly, Social Cognition were associated with depression levels measured by BDI total score and Somatic-Performance symptoms scores (BDI SP-Sentence Completion: *r* = –0.22, *p* = 0.019; Social Cognition: *r* = –0.29, *p* = 0.007; BDI Total score-Sentence Completion: *r* = –0.20, *p* = 0.023; Social Cognition: *r* = –0.26, *p* = 0.016). Mild correlations were also observed between Cognitive-Affective BDI subscore and Social Cognition (*r* = –0.19, *p* = 0.028). Moreover, BDI Total Score negatively correlated with performance at the Spelling test (BDI Total score-Spelling: *r* = –0.20, *p* = 0.028). Finally, significant but weak correlations were also observed between depression scores and Immediate Recall subtest within the Memory subdomain (BDI SP – Immediate recall: *r* = –0.16, *p* = 0.044; BDI Total Score-Immediate Recall: *r* = –0.18, *p* = 0.029).

### Relationship of Psychological Symptoms to Performance at Standard Cognitive Screening Tools

No significant correlations were observed between depression assessment (BDI), concerning both total, cognitive-affective and somatic-performance symptoms, and traditional screening of frontal abilities (FAB) and global cognition (MoCA). Similarly, anxiety assessment (STAI-Y) did not correlated with either FAB or MoCA for neither state or trait anxiety scores.

## Discussion

This study revealed the presence of mild significant correlations between depression scores and the ECAS, mainly concerning the executive functions subdomain. Such findings are in accordance with previous literature regarding patients with depression without neurological illness. In particular, some recent reviews and meta-analysis about cognitive impairment in depressed patients showed that depression is related to reduction in a wide range of cognitive abilities, including attention, processing speed, executive function, and memory (Lim et al., 2013; Bortolato et al., 2014; Ahern and Semkovska, 2016); in particular, larger impairments were observed in executive abilities concerning processing speed and shifting, while memory scores were affected by small to moderate impairments (Ahern and Semkovska, 2016). A recent review on adult individuals with depression focusing on studies employing neuropsychological tests for executive functions showed that the majority of studies included (25 of 28) found alterations in some aspects of executive functioning in patients with depression (Alves et al., 2014). Moreover, deficit in executive functions and attention seem to persist after remission of depressive symptoms, in particular with regard to inhibition, shifting and verbal fluency (Rock et al., 2014; Ahern and Semkovska, 2016).

In our sample, more detailed analysis within the executive functions subdomain revealed a more significant involvement of social cognition abilities in association with depression scores. Such finding is in agreement with literature underling a positive relationship between severity of depression symptoms and degree of Theory of Mind (ToM) impairments in depressed patients (Cusi et al., 2013; Bora and Berk, 2016). In a recent meta-analysis, ToM deficits were not influenced by severity of executive dysfunctions, suggesting that social cognition impairment could represent a separate domain affected in depressive disorders, aside with executive functions (Bora and Berk, 2016). Debate is still open about the source of social cognition deficits in ALS as studies provide heterogeneous results about their independence from executive dysfunction (Consonni et al., 2016; Strong et al., 2017). Another possible explanation is the one considering the inhibitory component as specifically associated to depression symptoms in ALS, resembling what described in depressed patients without neurological illness (Alves et al., 2014). In our sample, such consideration is supported by the association between depression scores and tasks markedly involving inhibitory abilities, i.e., Sentence Completion and Social Cognition within the Executive Functions subdomain.

Weak correlations have been observed in our sample between memory subdomain, in particular the Immediate Recall test, and depression scores. The involvement of memory functions, in particular immediate recall, cannot be clearly separated by the influence of attention and executive alterations, since available cognitive tests, and also those included into the ECAS, are usually related to both cognitive domains. In ALS population, the lack of consensus about the characterization of memory alterations and the poor specificity of such components leaded to not include them in the current criteria for ALS-FTD (Strong et al., 2017). Therefore, the collected data do not actually support an association between depression and memory function in our sample, also according to the weak correlations recorded.

Mild correlations have also been observed between the Spelling test within the language subdomain and depression score. Such data, not confirmed by available literature, needs further investigations and more significant data to be more critically considered.

Only few studies have investigated the association between depression symptoms and cognition in pALS. With regard to available literature in this field, our data are globally in accordance with two previous studies (Jelsone-Swain et al., 2012; Wei et al., 2016), highlighting an effect of depressive symptoms on cognitive performance at both global and specific neuropsychological measures in ALS. Conversely, contrasting results have been observed in other previous studies employing both traditional and motor-verbal free cognitive testing, showing the absence of a relationship between cognitive impairment and depression levels (Poletti et al., 2016a,b; Rabkin et al., 2016). The novelty of such topic in ALS, in association with the variety of measures for depression and cognition employed and the different modes of administration of neuropsychological tests (both ‘paper and pencil’ and motor-verbal free based measures), makes a comparison with previous findings not feasible. Possibly, the increasing adoption of a recently validated gold standard measure for cognition and behavior in ALS, i.e., the ECAS, will allow to provide comparable data across studies and to realize longitudinal investigations, due to the partial compensation of motor disability. Moreover, the absence of correlations observed between depression scores and performance at standard cognitive screening tools (FAB and MoCA) further support the use of the ECAS for the investigation of the discussed topic according to both sensitivity and feasibility components of such instrument in ALS. In our sample, association with cognition mainly concerned Total BDI Score and Somatic-Performance BDI components, while Cognitive-Affective components were only poorly associated with cognitive scores. These results could be controversial, according to doubts that BDI may overestimate the presence of depression in ALS, since it contains a number of somatic/vegetative symptoms that can overlap with physical illness. However, the use of BDI in our protocol is consistent with previous studies that confirmed the reliability of such measure in pALS. A previous study comparing the estimated prevalence of depression using BDI and a questionnaire specifically designed for pALS revealed little discrepancy between findings (Kubler et al., 2005). Also the use of a modified BDI scale, without items that could be confounded by physical symptoms, showed good agreement with the standard BDI scale (Wicks et al., 2007). Therefore, the BDI score can be considered appropriate for clinical use in ALS population (Kubler et al., 2005; Wicks et al., 2007; Taylor et al., 2010).

In depressed patients, both somatic/vegetative symptoms and negative affective states/dysfunctional cognitive patterns may interfere with optimal performance. According to our results, we could hypothesize that in pALS the influence of depression over cognition, in particular social cognition abilities, may be more specifically mediated by somatic/vegetative symptoms. If confirmed by further studies, this result could be discussed within the frame of psychophysiological changes, involving the sympathetic nervous system, observed in ALS in association to social and emotional impairments (Lulé et al., 2005).

With regard to another psychological components investigated, i.e., state and trait anxiety, we found not significant associations with cognitive performances at the ECAS. According to recent literature, experimentally induced anxiety seems to impair performance only under low-load, i.e., simple cognitive tasks, while its effect is reduced when subjects engage in more difficult tasks that involve high attentional and executive resources (Vytal et al., 2012, 2013). Previous results obtained in ALS underlined an effect of anxiety on reasoning times, with higher levels of anxiety corresponding to lower execution times at some cognitive tests (Poletti et al., 2016a,b). In our sample, the limited proportion of patients presenting with clinically relevant state and trait anxiety levels, with global mean scores considerably lower than cut off, could explain the absence of clear effects of such psychological component over the observed performances. Moreover, previously reported associations between anxiety and execution times would not have been observed with the employed protocol that not include time-related measures. Therefore, further investigations are needed in ALS in order to clarify such issue.

Limitations of the present study mainly concern the psychological evaluation of pALS. In particular, informations about previous depressive episodes that could have helped in distinguishing between recurrent depressive episodes and depressive symptoms reactive to ALS diagnosis have not been systematically collected and described. Moreover, exclusion of patients taking psychotropic drugs, or distinction of such subgroup for data analysis, would have been useful to prevent or highlight the potential effect of medications on cognitive performances.

Even if assessed in a large sample of pALS, the relationship between psychological factors and executive functions in such population cannot be considered fully clarified. Probably, the investigation about the complexity of factors modulating executive functions will benefit from the fully control of psychological variables, verbal-motor components of cognitive tests and other well-known aspects influencing cognitive performances (i.e., socio-demographic variables).

Despite the above mentioned limitations, if confirmed by further investigations and by stronger statistical results, the observed association between depression and executive functions could help to better plan both cognitive assessment/training and psychological interventions in ALS. With regard to the former, to emphasize the importance of a careful screening for depression could help to avoid a cognitive involvement overestimation. Furthermore, the observed correlation could mask the progression of cognitive involvement as measured in longitudinal assessments. Such point should be targeted in future follow-up studies with the ECAS. With regard to psychological interventions, the explanation about how comorbid cognitive impairment, as well as other biopsychosocial factors, moderate both pharmacotherapy and psychotherapy outcomes in ALS could turn into more tailored and effective interventions. Actually, due to the poor definition of the influence between cognitive and psychological aspects in ALS, cognitive alterations are mostly not considered in psychological interventions in ALS (Gould et al., 2015). Additionally, the influence of psychotherapy on cognitive aspects of pALS should be more deeply investigated, according to recent literature showing an association between changes in the central nervous systems, i.e., an attenuation in disease progression, and a concomitant psychological treatment (Kleinbub et al., 2015).

The presented work could represent an important step toward the definition of a integrated approach to intervention for pALS, in accordance with the biopsychosocial model, with the aim to carefully and more efficiently manage such complex disease.

## Author Contributions

BP, LC, and FS conceived the study and wrote the manuscript. AL, SZ, and AM administered the cognitive and psychological protocols. AF and FM performed the statistical analysis. AC, AD, and NT performed the clinical examination of ALS patients. BP and VS critically revised the manuscript.

## Conflict of Interest Statement

The authors declare that the research was conducted in the absence of any commercial or financial relationships that could be construed as a potential conflict of interest. The reviewer AP and handling Editor declared their shared affiliation.

## References

[B1] Abrahams S. Newton J. Niven E. Foley J. Bak T. (2014). Screening for cognition and behaviour changes in ALS. *Amyotroph. Lateral Scler. Frontotemporal Degener.* 15 9–14. 10.3109/21678421.2013.805784 23781974

[B2] Agosta F. Ferraro P. M. Riva N. Spinelli E. G. Chiò A. Canu E. (2016). Structural brain correlates of cognitive and behavioral impairment in MND. *Hum. Brain Mapp.* 37 1614–1626. 10.1002/hbm.23124 26833930 PMC6867462

[B3] Ahern E. Semkovska M. (2016). Cognitive functioning in the first-episode of major depressive disorder: a systematic review and meta-analysis. *Neuropsychology* 31 52–72. 10.1037/neu0000319 27732039

[B4] Alves M. R. Yamamoto T. Arias-Carrión O. Rocha N. B. F. Nardi A. E. Machado S. (2014). Executive function impairments in patients with depression. *CNS Neurol. Disord. Drug Targets* 13 1026–1040. 10.2174/1871527313666140612102321 24923347

[B5] Beck A. T. Ward C. H. Mendelson M. Mock J. Erbaugh J. (1961). An inventory for measuring depression. *Arch. Gen. Psychiatry* 4 561–571. 10.1001/archpsyc.1961.01710120031004 13688369

[B6] Benjamini Y. Hochberg Y. (1995). Controlling the false discovery rate: a practical and powerful approach to multiple testing. *J. R. Stat. Soc. Ser. B* 57 289–300.

[B7] Bora E. Berk M. (2016). Theory of mind in depressive disorder: a meta-analysis. *J. Affect. Disord.* 191 49–55. 10.1016/j.jad.2015.11.023 26655114

[B8] Bortolato B. Carvalho A. F. McIntyre R. S. (2014). Cognitive dysfunction in major depressive disorder: a state-of-the-art clinical review. *CNS Neurol. Disord._Drug Targets* 13 1804–1818. 10.2174/1871527313666141130203823 25470396

[B9] Brakowski J. Spinelli S. Dörig N. Bosch O. G. Manoliu A. Holtforth M. G. (2017). Resting state brain network function in major depression - Depression symptomatology, antidepressant treatment effects, future research. *J. Psychiatr. Res.* 92 147–159. 10.1016/j.jpsychires.2017.04.007 28458140

[B10] Brooks B. R. Miller R. G. Swash M. Munsat T. L. World Federation of neurology Research Group on Motor Neuron Disease. (2000). El Escorial revisited: revised criteria for the diagnosis of amyotrophic lateral sclerosis. *Amyotroph. Lateral Scler Other Motor Neuron Disord.* 1 293–299. 10.1080/146608200300079536 11464847

[B11] Cedarbaum J. M. Stambler N. Malta E. Fuller C. Hilt D. Thurmond B. (1999). The ALSFRS-R: a revised ALS functional rating scale that incorporates assessment of respiratory function. *J. Neurol. Sci.* 169 13–21. 10.1016/S0022-510X(99)00210-5 10540002

[B12] Consonni M. Catricalà E. Dalla Bella E. Gessa V. C. Lauria G. Cappa S. F. (2016). Beyond the consensus criteria: multiple cognitive profiles in amyotrophic lateral sclerosis? *Cortex* 1 162–167. 10.1016/j.cortex.2016.04.014 27236371

[B13] Conti S. Bonazzi S. Laiacona M. Masina M. Vanelli Coralli M. (2014). Montreal cognitive assessment (MoCA)-Italian version: regression based norms and equivalent scores. *Neurol. Sci.* 36 209–214. 10.1007/s10072-014-1921-3 25139107

[B14] Cusi M. A. Nazarov A. MacQueen G. M. McKinnon M. C. (2013). Theory of mind deficits in patients with mild symptoms of major depressive disorder. *Psychiatry Res.* 210 672–674. 10.1016/j.psychres.2013.06.018 23850430

[B15] DeJesus-Hernandez M. Mackenzie I. R. Boeve B. F. Boxerl A. L. Baker M. Rutherford N. J. (2011). Expanded GGGGCC hexanucleotide repeat in noncoding region of C9ORF72 causes chromosome 9p-linked FTD and ALS. *Neuron* 72 245–256. 10.1016/j.neuron.2011.09.011 21944778 PMC3202986

[B16] Dubois B. Slachevsky A. Litvan I. Pillon B. (2000). The FAB. A frontal assessment battery at bedside. *Neurology* 55 1621–1626. 10.1212/WNL.55.11.1621 11113214

[B17] Elamin M. Bede P. Byrne S. Jordan N. Gallagher L. Wynne B. (2013). Cognitive changes predict functional decline in ALS: a population-based longitudinal study. *Neurology* 80 1590–1597. 10.1212/WNL.0b013e31828f18ac 23553481

[B18] Goldstein L. H. Abrahams S. (2013). Changes in cognition and behaviour in amyotrophic lateral sclerosis: nature of impairment and implications for assessment. *Lancet Neurol.* 12 368–380. 10.1016/S1474-4422(13)70026-7 23518330

[B19] Gould R. L. Coulson M. C. Brown R. G. Goldstein L. H. Al-Chalabi A. Howard R. J. (2015). Psychotherapy and pharmacotherapy interventions to reduce distress or improve well-being in people with amyotrophic lateral sclerosis: a systematic review. *Amyotroph. Lateral Scler. Frontotemporal Degener.* 16 293–302. 10.3109/21678421.2015.1062515 26174444

[B20] Jelsone-Swain L. Persad C. Votruba K. L. Weisenbach S. L. Johnson T. Gruis K. L. (2012). The relationship between depressive symptoms, disease state, and cognition in amyotrophic lateral sclerosis. *Front. Psychol.* 3:542. 10.3389/fpsyg.2012.00542 23411492 PMC3571885

[B21] Kleinbub J. R. Palmieri A. Broggio A. Pagnini F. Benelli E. Sambin M. (2015). Hypnosis-based psychodynamic treatment in ALS: a longitudinal study on patients and their caregivers. *Front. Psychol.* 6:822. 10.3389/fpsyg.2015.00822 26136710 PMC4469765

[B22] Kubler A. Winter S. Ludolph A. C. Hautzinger M. Birbaumer N. (2005). Severity of depressive symptoms and quality of life in patients with amyotrophic lateral sclerosis. *Neurorehabil. Neural Repair* 19 182–193. 10.1177/1545968305276583 16093409

[B23] Kurt A. Nijboer F. Matuz T. Kübler A. (2007). Depression and anxiety in individuals with amyotrophic lateral sclerosis: epidemiology and management. *CNS Drugs* 21 279–291. 10.2165/00023210-200721040-00003 17381183

[B24] Lim J. Oh I. K. Han C. Huh Y. J. Jung I. K. Patkar A. A. (2013). Sensitivity of cognitive tests in four cognitive domains in discriminating MDD patients from healthy controls: a meta-analysis. *Int. Psychogeriatr.* 25 1543–1557. 10.1017/S1041610213000689 23725644

[B25] Lulé D. Burkhardt C. Abdulla S. Böhm S. Kollewe K. Uttner I. (2015). The Edinburgh cognitive and behavioural amyotrophic lateral sclerosis screen: a cross-sectional comparison of established screening tools in a German-Swiss population. *Amyotroph. Lateral Scler. Frontotemporal Degener.* 16 16–23. 10.3109/21678421.2014.959451 25292386

[B26] Lulé D. Kurt A. Jürgens R. Kassubek J. Diekmann V. Kraft E. (2005). Emotional responding in amyotrophic lateral sclerosis. *J. Neurol.* 252 1517–1524. 10.1007/s00415-005-0907-8 15977000

[B27] Montuschi A. Iazzolino B. Calvo A. Moglia C. Lopiano L. Restagno G. (2015). Cognitive correlates in amyotrophic lateral sclerosis: a population-based study in Italy. *J. Neurol. Neurosurg. Psychiatry* 86 168–173. 10.1136/jnnp-2013-307223 24769471

[B28] Niven E. Newton J. Foley J. Colville S. Swingler R. Chandran S. (2015). Validation of the Edinburgh cognitive and behavioural amyotrophic lateral sclerosis screen (ECAS): a cognitive tool for motor disorders. *Amyotroph. Lateral Scler. Frontotemporal Degener.* 16 172–179. 10.3109/21678421.2015.1030430 25967542

[B29] Nunnari D. De Cola M. C. D’Aleo G. Rifici C. Russo M. Sessa E. (2015). Impact of depression, fatigue, and global measure of cortical volume on cognitive impairment in multiple sclerosis. *Biomed. Res. Int.* 2015:519785. 10.1155/2015/519785 25861633 PMC4377448

[B30] Phukan J. Elamin M. Bede P. Jordan N. Gallagher L. Byrne S. (2012). The syndrome of cognitive impairment in amyotrophic lateral sclerosis: a population-based study. *J. Neurol. Neurosurg. Psychiatry* 83 102–108. 10.1136/jnnp-2011-300188 21836033

[B31] Pirani A. Nasreddine Z. S. Tulipani C. Neri M. (2007). “Montreal cognitive assessment (MOCA): uno strumento rapido per lo screening del mild cognitive impairment. Dati preliminari della versione Italiana,” in *Atti IV Congresso Regionale Associazione Italiana Psicogeriatria*, Bologna.

[B32] Poletti B. Carelli L. Solca F. Lafronza A. Pedroli E. Faini A. (2016a). An eye-tracker controlled cognitive battery: overcoming verbal-motor limitations in ALS. *J. Neurol.* 264 1136–1145. 10.1007/s00415-017-8506-z 28503706

[B33] Poletti B. Carelli L. Solca F. Lafronza A. Pedroli E. Faini A. (2016b). Cognitive assessment in Amyotrophic Lateral Sclerosis by means of P300-brain computer interface: a preliminary study. *Amyotroph. Lateral Scler. Frontotemporal Degener.* 7 473–481. 10.1080/21678421.2016.1181182 27169693

[B34] Poletti B. Solca F. Carelli L. Madotto F. Lafronza A. Faini A. (2016c). The validation of the Italian Edinburgh cognitive and behavioural ALS screen (ECAS). *Amyotroph. Lateral Scler. Frontotemporal Degener.* 17 489–498. 10.1080/21678421.2016.1183679 27219526

[B35] Pu S. Setoyama S. Noda T. (2017). Association between cognitive deficits and suicidal ideation in patients with major depressive disorder. *Sci. Rep.* 7:11637. 10.1038/s41598-017-12142-8 28912439 PMC5599636

[B36] Rabkin J. Goetz R. Murphy J. M. Factor-Litvak P. Mitsumoto H. Als Cosmos Study Group. (2016). Cognitive impairment, behavioral impairment, depression, and wish to die in an ALS cohort. *Neurology* 87 1320–1328. 10.1212/WNL.0000000000003035 27496520 PMC5573192

[B37] Radakovic R. Stephenson L. Newton J. Crockford C. Swingler R. Chandran S. (2017). Multidimensional apathy and executive dysfunction in Amyotrophic lateral Sclerosis. *Cortex* 94 142–151. 10.1016/j.cortex.2017.06.023 28759804

[B38] Rock P. L. Roiser J. P. Riedel W. J. Blackwell A. D. (2014). Cognitive impairment in depression: a systematic review and meta-analysis. *Psychol. Med.* 44 2029–2040. 10.1017/S0033291713002535 24168753

[B39] Snowden M. B. Atkins D. C. Steinman L. E. Bell J. F. Bryant L. L. Copeland C. (2015). Longitudinal association of dementia and depression. *Am. J. Geriatr. Psychiatry* 23 897–905. 10.1016/j.jagp.2014.09.002 25441056 PMC4369182

[B40] Spielberger C. D. Gorsuch R. L. Lushene R. E. (1970). *The State-Trait Anxiety Inventory(test manual)*. Palo Alto, CA: Consulting Psychologists Press.

[B41] Strong M. J. Abrahams S. Goldstein L. H. Wolley S. Mclaughlin P. Snowden J. (2017). Amyotrophic lateral sclerosis - frontotemporal spectrum disorder (ALS-FTSD): revised diagnostic criteria. *Amyotroph. Lateral Scler. Frontotemporal Degener.* 18 153–174. 10.1080/21678421.2016.1267768 28054827 PMC7409990

[B42] Taylor L. Wicks P. Leigh P. N. Goldstein L. H. (2010). Prevalence of depression in amyotrophic lateral sclerosis and other motor disorders. *Eur. J. Neurol.* 17 1047–1053. 10.1111/j.1468-1331.2010.02960.x 20158515

[B43] Terroni L. Sobreiro M. F. M. Conforto A. B. Adda C. C. Guajardo V. D. de Lucia M. C. S. (2012). Association among depression, cognitive impairment and executive dysfunction after stroke. *Dement Neuropsychol.* 6 152–157. 10.1590/S1980-57642012DN06030007 29213789 PMC5618962

[B44] van Groenestijn A. C. Kruitwagen-van Reenen E. T. Visser-Meily J. M. A. van den Berg L. H. Schröder C. D. (2016). Associations between psychological factors and health-related quality of life and global quality of life in patients with ALS: a systematic review. *Health Qual. Life Outcomes* 14:107. 10.1186/s12955-016-0507-6 27439463 PMC4955215

[B45] Vytal K. Cornwell B. Arkin N. Grillon C. (2012). Describing the interplay between anxiety and cognition: from impaired performance under low cognitive load to reduced anxiety under high load. *Psychophysiology* 49 842–852. 10.1111/j.1469-8986.2012.01358.x 22332819 PMC3345059

[B46] Vytal K. Cornwell B. Letkiewicz A. Arkin N. Grillon C. (2013). The complex interaction between anxiety and cognition: insight from spatial and verbal working memory. *Front. Hum. Neurosci.* 7:93. 10.3389/fnhum.2013.00093 23542914 PMC3610083

[B47] Wei Q. Chen X. Cao B. Ou R. Zhao B. Wu Y. (2016). Associations between neuropsychiatric symptoms and cognition in Chinese patients with amyotrophic lateral sclerosis. *Amyotroph. Lateral Scler. Frontotemporal Degener.* 17 358–365. 10.3109/21678421.2016.1154574 26962892

[B48] Wicks P. Abrahams S. Masi D. Hejda-Forde S. Leigh P. N. Goldstein L. H. (2007). Prevalence of depression in a 12-month consecutive sample of patients with ALS. *Eur. J. Neurol.* 14 993–1001. 10.1111/j.1468-1331.2007.01843.x 17718691

[B49] Xu Z. Alruwaili A. R. S. Henderson R. D. McCombe P. A. (2017). Screening for cognitive and behavioural impairment in amyotrophic lateral sclerosis: frequency of abnormality and effect on survival. *J. Neurol. Sci.* 376 16–23. 10.1016/j.jns.2017.02.061 28431606

[B50] Yoon S. Shin C. Han C. (2017). Depression and cognitive function in mild cognitive impairment: a 1-year follow-up study. *Geriatr. Psychiatry Neurol.* 30 280–288. 10.1177/0891988717723741 28925333

